# Drunkenness a Disease

**Published:** 1871-03

**Authors:** 


### DRUNKENNESS A DISEASE.

UNDER this view of the case it is said that more than half the persons who submit to the treatment are cured in the hands of Dr Dodge, at the New York State Asylum for Inebriates at Binghampton, Dr. Parsish of Philadelphia, and Dr. Day. The method of treatment adopted by Dr. Dodge, a gentleman of wealth and culture and professional ability, is to remove the cause promptly and completely on entering the establishment, then to gain the coöperation and confidence of the patient. Such food is provided for each as is calculated to tempt the appetite and promote digestion, and thus bring back the power of a debilitated and outraged stomach; for it is a good digestion which gives strength of body as well as a vigorous and healthy working brain, which of itself is a great aid to strong resolutions not to touch, taste, or smell the abominable thing. To give varied occupation to the mind and exercise to the body, with a motive, a great variety of amusements are inaugurated, — as billiards, chess, backgammon, dominoes, and out-door sports, with a well-appointed reading-room, and a variety of social gatherings, reunions, and conversations ; then there are pianos, organs, violins, banjos, harps, and vocal music besides. When patients have been there long enough to acquire force of will sufficient to enable them to resist any ordinary temptation, they are allowed to range over field and wood, and employ themselves in cutting canes, making curious toys, and contriving devices for suggestion and amusement. Everything done is of a nature calculated to elevate, refine, and ennoble.

This is the bright side. We believe that drunkenness is a habit, an appetite cultivated to the extent of becoming inappeasible, like a natural passion, and which will not be satisfied without an indulgence, to return again forever, like the sense of hunger for food, or thirst for water; like the passions of our nature. Now and then a man may have such an extraordinary force of will as to overpower the passion, just as some men have force of will enough to resist eating until they die. But such powers of will are not found oftener than once in a thousand. The only safety from a ruinous life, a disgraced name, and a beastly death, is the systematic, habitual avoidance of spirituous liquors in all their forms, from cider and lager beer to wine, brandy, and absinthe; for the use of the mildest beverage now and then, in courtesy to friend or hostess, is but the first step to a ruined fortune, and name, and body, and soul; and to avoid these first steps to death, so disgraceful and inevitable, is to make home the pleasantest place in the world for children, so as to keep them out of the city street, and away from the country grocery, the circus, the theatre, and railroad station. Train the children early to the conviction that they must do something for a living; teach them that to earn money honestly is a first virtue, and next to that saving it; any child practicing these once, is saved for all time.

